# Early life predictors of preschool overweight and obesity: a case–control study in Sri Lanka

**DOI:** 10.1186/1471-2458-13-994

**Published:** 2013-10-22

**Authors:** Kumari M Rathnayake, Aberna Satchithananthan, Senarath Mahamithawa, Ranil Jayawardena

**Affiliations:** 1 Department of Applied Nutrition, Faculty of Livestock, Fisheries and Nutrition, Wayamba University of Sri Lanka, Makandura 60170, Sri Lanka; 2 Nutrition Division, Ministry of Health, Public Health Complex, Colombo 05, Sri Lanka; 3 Institute of Health and Biomedical Innovation, Faculty of Health, Queensland University of Technology, Brisbane, QLD 4059, Australia

**Keywords:** Preschool, Overweight, Obesity, Rapid weight gain

## Abstract

**Background:**

Childhood obesity increases the risk of obesity in adulthood and is associated with cardiovascular disease risk factors. Our aim was to assess the early life risk factors associated with overweight and obesity among preschool children.

**Methods:**

In this case–control study, from the 1087 preschool children measured, age, sex and ethnicity matched 71 cases and 71 controls were recruited. Cases and controls were defined according to the WHO 2006 growth standards. The birth and growth characteristics were extracted from the child health development records. Infant feeding practices and maternal factors were obtained from the mother. Rapid weight gain was defined as an increase in weight-for-age Z score (WHO standards) above 0.67 SD from birth to 2 years. The magnitude and significant difference in mean values of the variables associated with overweight and obesity were evaluated using logistic regressions and paired t-test, respectively.

**Results:**

Cases had significantly shorter duration (months) of breastfeeding (19.4, 24.6, p = 0.003), and smaller duration (months) of exclusive breastfeeding (3.7, 5.1, p = 0.001) compared to controls. Rapid weight gain (OR = 6.3, 95% CI = 2.04–19.49), first born status (OR = 3.6, 95% CI = 1.17-10.91) and pre-pregnancy obesity (OR = 4.0, 95% CI = 1.46-10.76) were positively associated with overweight and obesity. Breastfeeding more than 2 years (OR = 0.2, 95% CI = 0.06-0.57) was negatively associated with overweight and obesity.

**Conclusion:**

Rapid weight gain within first two years, first–born status and pre-pregnancy obesity of the mother contributed for preschool obesity. Our results suggest that intervention may be indicated earlier in infancy and during the toddler and preschool years to tackle the increasing prevalence of obesity.

## Background

Childhood obesity increases the risk of obesity in adulthood and is associated with cardiovascular disease risk factors such as hypertension, diabetes mellitus and dyslipidemia [1]. The increase in the prevalence of obesity in recent years has brought this condition to the forefront of the public health concern in many countries. The prevalence of obesity is increasing both in developed and developing countries and has become a major public health problem [2]. The roots of the obesity epidemic need to be tracked back as early in life as possible in order to develop effective means for preventing this condition and its health consequences in the future.

To prevent obesity, both the risk factors and the critical periods for development of obesity must be identified [3]. There is substantial evidence that childhood obesity proceeds into adulthood obesity, especially children of obese parents [4]. Gestation and early infancy, the period of adiposity rebound (5–7 years old) and adolescence have been identified as the critical period for the development of obesity [5]. Several maternal and birth factors have been studied extensively for their association with obesity in the latter part of their life. Large infant size in the first months of life has been associated with obesity in childhood [6,7]. Rapid weight gain which was traditionally considered as a healthy intervention for low birth weight infants is now recognized as a potential risk factor of increasing interest for obesity. A pattern of rapid weight gain during early life is associated with obesity not only in childhood, but also in young adulthood [3]. First born status, female sex and maternal body mass index (BMI) also have been identified as risk factors for the development of obesity in several studies [8].

Preliminary studies conducted in Sri Lanka have revealed that serious metabolic consequences of obesity are seen in Sri Lankan children [9]. However there are very few studies on the prevalence and the risk factors associated with preschooler overweight and obesity. The overweight and obesity prevalence in the age group of 0–5 years in South East Asia is 4.6% in 2010, and is expected to be increased to 6.7% in 2020 [10] whereas in Sri Lanka the prevalence of overweight (weight-for-height z score) among children under 5 years old is 1.6% according to the national demographic and health survey data [11].

Studies addressing the risk factors associated with obesity or overweight in Sri Lanka is limited in preschool age group. Therefore, the aim of our present study was to assess the early life risk factors associated with overweight and obesity among preschool children in the Sri Lankan scenario. The findings of this study will be useful in identifying effective interventions to combat the paediatric obesity.

## Methods

### Study design and participants

This was conducted as a case and control study comprising 71 cases and 71 controls of 3–5 years old children, to examine a broad range of factors related to preschooler’s overweight and obesity simultaneously and to determine which factors exert a significant influence in early life.

The cases (overweight and obese) and controls (normal children) were recruited from preschools. Among the 30 schools contacted, 16 gave the permission to conduct the study. The height and weight of all the children (n = 1087) in the 16 preschools were measured and their BMI–for–age Z score was calculated. BMI–for–age Z score above +2SD and between -2SD and +2SD were considered as cases and controls, respectively according to the WHO 2006 growth standards [12]. Age, sex and ethnicity matched cases and controls were recruited on a 1:1 ratio individually from the same school and class. Subject information sheet about the study and the consent form were sent to the recruited children’ parents through the class teachers and responses were collected.

### Data collection

An interviewer administered questionnaire was used to collect the information from the mother. The interview was conducted either in person or over the phone. Child Health Development Record (CHDR) and antenatal records of the mother were observed. CHDR is a child health record, maintained by the Public Health Midwives throughout the childhood and kept in the custody of the mother. Information regarding birth characteristics, maternal factors and feeding behaviors were obtained from the records and by interviewing the mothers.

### Birth characteristics

Information on birth weight, birth length, weight at 6, 9, 12 and 24 months and rapid weight gain from birth to 2 years old were obtained from the CHDR. All the children were born >37 weeks gestational period. The information about gestational period was available from the antenatal/pregnancy record. The birth weight was categorized into low birth weight (LBW), normal birth weight (NBW) and high birth weight (HBW) [13]. Infant weight gain during the first year and second year of life was calculated by subtracting the birth weight of the subject from the weight at 12 and 24 months, respectively. Rapid weight gain was defined as a greater than 0.67SD change in weight-for-age Z score from birth to 2 years old. This has been suggested in other studies [14,15], and can be clinically interpreted as crossing the percentile lines in the growth chart.

Mode of delivery and parity were obtained from the mother during the interview. Delivery was classified as vaginal and by cesarean. Parity was considered as number of pregnancies resulting in live birth or still birth and was categorized as the first born child and non first born child [13]. The child rearing pattern was classified into two such as, brought up by mother and others.

### Maternal characteristics

Maternal characteristics such as educational level, occupation and family history of diabetes mellitus (DM)/hypertension (HT)/dyslipidemia (DL) were obtained at the interview. Mother’s age during delivery, pre-pregnancy body mass index (BMI), history of abortion and presence of gestational diabetes mellitus were obtained from the pregnancy record. Pre-pregnancy BMI was classified into two groups as, obese (≥ 27.5 kgm^-2^) or non obese mother prior to pregnancy. Mother’s level of education was classified as primary, secondary and tertiary education [14]. Maternal age was categorized into four groups such as, <18, 18–24, 25–29 and ≥30 years [13]. Accordingly, based on the glycaemic status mother was grouped into mother with normal glucose tolerance (NGT) and gestational diabetes mellitus (GDM). Working status of the mother was classified as working mother and non working mother. Family history of Diabetes mellitus (DM)/hypertension (HT)/dyslipidemia (DL) was classified into single parent with DM/HT/DL, both parents with DM/HT/DL, < 2 grandparents with DM/HT/DL and ≥2 grandparents with DM/HT/DL.

### Feeding practices

The duration of breastfeeding (BF), exclusive breastfeeding (EBF), the age of introduction of complementary food (CF) and infant formula were obtained from the mother during the interview. The duration of any breastfeeding was categorized into BF for 1 year, BF for 2 years and BF above 2 years. EBF duration was categorized into EBF for 6 months and no EBF for 6 months. Initiation of CF was categorized into such as initiation of CF before 6 months and at or after 6 months. Introduction of formula feeds was classified into 3 categories such as, initiation of formula before 6 months, 6 months to 1 year and 1–2 years [16].

### Data analysis

The statistical analysis was carried out by using the SPSS 16.0 software. Single and multiple logistic regression analysis were performed to analyze the association between potential categorical factors such as the questions related to the maternal and birth characteristics. Paired t-test was performed for the continuous data such as birth weight at 6, 9, 12 and 24 months, feeding variables such as breastfeeding duration, exclusive breastfeeding duration, complementary feeding and formula feeding introduction, to find out any significant difference between the mean values of matched cases and controls.

The Odds ratio (OR) and its 95% confidence interval (CI) were also estimated and computed for each significant categorical factor and continuous data using binary logistic regression. A factor with an OR significantly (p < 0.05) higher than 1.00 was taken as a risk factor of childhood obesity, while OR significantly (p < 0.05) less than 1.00 was regarded as a protective factor. In the paired t-test analysis a factor with a p value < 0.05 was considered as significant and > 0.05 was considered as insignificant. The goodness of fit of the final logistic regression model was analyzed using the Hosmer-Lemeshow technique, in which a p value >0.05 indicates a good model. The nutritional status of the study population was determined from the WHO 2005 Anthro-software by entering the sex, date of birth, weight and height data.

The ethical clearance for this study was obtained from the ethical review committee of Sri Lanka Medical Association (ERC 12/030), and informed consent was obtained from the parents before the data was collected.

## Results

The study population consists of 44 males and 27 females of 3 – 5 years old in each group (cases and controls). The mean age of the study population was 4.2 ± 0.76 years (Table 1). The overall prevalence of overweight and obesity of the total study population (n = 1087) was 6.2% and 1.2%, respectively. The prevalence of obesity among boys and girls was 1.9% and 0.6%, respectively (Table 2).

**Table 1:** General characteristics of the study population

**Characteristics**		**Included in final analysis (n = 142)**
**Maternal**	Mean age (years ± SD)	34.6 ± 5.2
	Mean age at delivery (years ± SD)	29.3 ± 4.7
	***Education: n (%)***	
	Tertiary	85 (59.8%)
	Secondary	33 (23.2%)
	Primary	24 (17.0%)
	***Income***	
	0 – 50,000 LKR	68 (47.8%)
	>50,000 LKR	74 (52.2%)
**Child**	Mean birth weight (kg ± SD)	3.2 ± 0.4
	***Sex: n (%)***	
	Male	88 (62.0%)
	Female	54 (38.0%)
	Mean age at assessment (years ± SD)	4.2 ± 0.8

**Table 2:** Nutritional status of the study population (n = 1087)

	**Obesity**	**Overweight**	**Underweight**	**Wasting**	**Stunting**
Boys	1.9	8.2	8.0	10.8	5.9
Girls	0.6	4.3	9.4	10.9	6.2
Total	1.2	6.2	8.7	10.8	6.0

There was a significant difference in the birth characteristics such as weight at 6,9,12 and 24 months and weight gain up to 1 & 2 years between the matched cases and controls. There was a significant difference in the duration of breastfeeding and exclusive breast feeding (EBF) between the cases and controls, where the controls were having a longer duration of breast feeding and extended EBF compared to the cases (Table 3).

**Table 3:** Comparison of birth characteristics and feeding characteristics among cases and controls

**Factors**	**Cases**		**Controls**		**P value**^**a**^
	**Mean**	**SD**	**Mean**	**SD**	
Birth length (cm)	50.1	4.3	50.1	4.0	0.162
Birth weight (kg)	3.1	0.3	3.0	0.6	0.065
***Weight at (g)***					
6 months	7740	658.7	7335	733	**0.001**
9 months	9045	882	8394.4	843.6	**<0.0001**
12 months	10100	931.5	9013	860	**<0.0001**
24 month	13500	1545	11000	1150	**<0.0001**
***Weight gain upto (g)***					
1 year old	6882	985.3	5761.4	843.2	**<0.0001**
2 years old	10300	1474	7804	1067	**<0.0001**
***Feeding characteristics***					
Duration of breastfeeding	19.4	13.6	24.6	16.2	**0.041**
EBF duration	**3.8**	2.5	**5.0**	2.7	**0.003**
Formula initiation	5.5	6.0	5.4	4.3	0.904
Complementary food introduction	5.8	0.9	6.5	2.3	**0.026**

^a^values were obtained from paired-t test. Significant p values are given in bold type.

Following the literature, factors such as mother’s educational level, mother’s age during delivery, working status, presence of GDM, past history of abortion, obesity prior to pregnancy, child rearing pattern, family history of DM/HT/DL, birth weight, mode of delivery, parity, rapid weight gain, breastfeeding duration, EBF, complementary feeding initiation and formula feeding were considered for the univariable logistic regression among them, the factors which indicated significant relationship with overweight and obesity were carried forward for the multiple logistic regression (Table 4).

**Table 4:** Factors associated with preschool overweight and obesity (n = 142)

**Factors**	**Univariable analysis**		**Multivariable analysis**		
	**OR**^**b**^	**P value**^**b**^	**OR**^**b**^	**P value**^**a,b**^	**95% confidence interval**^**b**^
Mother with a degree	9.56	0.0001	2.12	0.276	0.681 – 6.614
Working mother	3.87	0.001	1.44	0.677	0.258 – 8.030
Childcare by mother	0.24	0.001	0.54	0.477	0.101 – 2.914
Rapid weight gain	6.46	0.0001	**6.29**	**0.001**	**2.036 – 19.49**
Cesarean delivery	2.66	0.007	0.70	0.518	0.248 – 2.02
Past history of abortion	0.16	0.0001	0.40	0.202	0.098– 1.634
First born status	5.32	0.0001	**3.57**	**0.025**	**1.169 – 10.91**
Pre-pregnancy BMI	2.39	0.018	**3.96**	**0.007**	**1.458 – 10.76**
Single parent with DM/HT/DL	2.35	0.039	3.30	0.051	0.995 – 10.95
>2 grandparents with DM/HT/DL	2.81	0.010	1.74	0.345	0.550 – 5.52
Breastfeeding above 2 years old	0.40	0.021	**0.18**	**0.004**	**0.057 – 0.571**
EBF for 6 month	0.33	0.002	0.80	0.686	0.283 – 2.297
Initiation of Complementary feeding after 6 months	0.30	0.046	0.33	0.149	0.072 – 1.494

^a^Significant p values (<0.05), OR and CI derived from final model are given in bold type.

^b^The confidence intervals, p values and OR were obtained from binary logistic regression analysis.

OR, Odds ratio; DM/HT/DL; diabetes mellitus/hypertension)/dyslipidemia, EBF; exclusive breast feeding.

Mother with a higher educational status, working mother, being obese prior to pregnancy, at least one parent and more than two grandparents having a history of DM/HT/DL, being the first born child in the family and rapid weight gain from birth to two years old were the potential risk factors individually influencing the overweight or obese conditions of a preschool child. The child reared by the mother, continuing breastfeeding upto 2 years old and beyond, initiating complementary feeding at or after 6 months and exclusive breastfeeding for 6 months in line with the WHO recommendations were found to be protective against preschool overweight and obesity when considering their effects individually (Table 4).

According to the multiple logistic regression analysis, the preschool child has higher chances of being overweight or obese, when he or she was the first child in the family and also when his/her mother was obese prior to pregnancy. Rapid weight gain during infancy, which is of increasing interest, has been found out as an important risk factor for preschool overweight and obesity in our study. Breastfeeding for more than 2 years old reduced the risk of preschool overweight and obesity (Table 4).

Hosmer-Lemeshow technique was used to assess the goodness of fit of the model, which indicates the extent to which the model provides better fit than a null model with no predictors. The test provided a p-value of 0.077 at 95% confidence interval which indicates it is a well-fitting model.

## Discussion

The identification of potential risk factors of young child obesity has been given much attention in the recent years. Many previous studies have been published to identify the risk factors of preschool obesity. Feeding characteristics [16], rapid weight gain in the first months of life [14], maternal and family history of obesity [16] have been identified as risk factors for obesity in previous studies.

It is particularly difficult to compare these results with ours, since they came from different study populations, each of which had its own geographical, cultural and behavioral determinants. The definition of young child obesity was also different in previous studies, which used a height adjusted weight for 120% of the NCHS mean [17] or BMI ≥ 90th percentile of French reference curves as cutoffs [18]. Present study used the BMI– for–age cutoff values for preschool children as recommended in the WHO 2006 standards. Therefore, this study was conducted with the aim of identifying the maternal and child risk factors associated with preschool overweight and obesity, considering several factors simultaneously.

The prevalence of overweight and obesity of the whole study population of 1087 children was 6.2% and 1.2%, respectively. The prevalence of overweight and obesity was prominent among males with 8.2% and 1.9%, respectively, while more females were underweight with a prevalence of 9.4%. According to the recent national demographic and health survey data [11], the prevalence of overweight among the children less than five years old was 1.6%, where males had a slightly higher prevalence (1.6%), compared to females (1.5%). This result is consistent with the other studies done in Sri Lanka, in the other age groups. In a study done with primary school children in the Colombo district of Sri Lanka, a prevalence of 5.1% obese and 8.9% overweight has been reported, where obese and overweight was more prominent among boys and underweight among girls [19].

The association of parental obesity with overweight among children has been reported consistently. It has previously been shown that pre-pregnant BMI in mothers is associated with obesity in young adulthood [20] but it has not been clear how early in life children who are born to obese mothers begin to express their risk for obesity. It has been demonstrated that this risk relationship does not emerge until at least 3–4 years of age [21]. In our study, the children who were born to obese mothers had higher risk of being overweight/obese at preschool age even after controlling for other factors in multiple logistic regression analysis. There are many mechanisms by which a mother’s obesity in early pregnancy might confer risk of obesity to her child, including the child’s inheritance of genes that confer susceptibility to obesity, the effects of maternal obesity on the intrauterine environment and the maternal role in shaping the child’s postnatal eating and activity environment [13].

The association of childhood obesity with growth indicators at birth and early childhood is of growing interest. Stettler *et al.,*[20] for the first time suggested that rapid weight gain from birth to the age of 4 months was associated with obesity in young adulthood. Our finding, rapid weight gain during the period from birth to two years old was associated with preschool overweight and obesity. A cohort study has explained this issue as rapid infancy weight gain was associated with leptin resistance, one of the mechanism thought to underlie obesity [20]. This child risk factor was significant even after adjusting for the other risk factors in the final model. Infants who grew more rapidly (usually measured as rapid weight gain) were more likely to be obese in childhood, adolescence or early adulthood than other infants. A UK cohort study done in children under 5 years old has evidenced the same trend that catch up growth (+0.67 SD increase in weight for age Z score) is associated with obesity in childhood with higher adjusted odds [15].

In this preschool study, the first born status of the child was individually associated with higher risk of overweight and obesity at preschool age. Significant number of studies has examined the independent effect of parity [22]. The first born status of the child is associated with increased risk of overweight at 2, 3 and 4 years old [13]. Previous findings suggest that although first born offspring are lighter at birth, being first born is associated with increased fat mass in both childhood and adolescence. The mechanisms are unknown, but experimental data report resetting of the leptin and glucocorticoid axis within the adipocyte, contributing to increased adipogenesis during late gestation and continuing after birth. This may contribute to the obesity epidemic in communities where there are restrictions in family size and a generation with greater proportion of first-born children [22].

In this current study breastfeeding for more than two years of age, exclusive breastfeeding for six months and initiation of complementary feeding at or after 6 months have been identified as protective factors against preschool overweight and obesity. However, only breastfeeding for above 2 years old remained significant when controlled for the other factors, with reduced risk for preschool overweight and obesity. Similar evidence was shown in an Indian study where they suggest that longer breastfeeding duration and later introduction of solid foods has a small reduction on later high BMI risk [23]. Breastfeeding has been associated with lower concentration of the appetite-regulating hormone, leptin in adolescents, raising the possibility that relative undernutrition and slower growth associated with breastfeeding in the first few weeks permanently programmes a lower appetite [24]. This may be the mechanism for which breastfed infants having low potential of being obese throughout their lifecycle.

Some limitations of the study must be considered in the interpretation of the findings. The sample size could have been expanded more, so that stronger association of risk factors could have been demonstrated. Sine this is a case and control study, certain risk factors would have been reversed during the time of data collection as the condition is known. Therefore the true relationship of certain current factors could have been reduced. Preschooler obesity is increasing in Sri Lanka and world over due to several modifiable factors. Several risk factors such as rapid weight gain, maternal obesity and feeding practices and their effect on obesity should be exclusively studied in order to design local prevention efforts.

## Conclusion

In conclusion, the current case control study indicates that pre-pregnancy obesity, being the first born baby of the particular family and rapid weight gain from birth to two years old were contributory factors for preschool overweight and obesity. Continuing breastfeeding above two years old was found to be protective. Prevention of overweight and obesity in children is generally focused on change in lifestyle during later childhood or adolescence. Our results indicate that early intervention may be more useful in infancy and during the toddler and preschool years to combat and control the increasing prevalence of obesity.

## Competing interests

The authors declare that they have no competing of interests.

## Authors’ contributions

KMR participated in the design of the study, data interpretation and drafted the manuscript. AS contributed to the data collection, data analysis, data interpretation and coordination of the study. SM participated in the design, critically revision of the manuscript and coordination of the study. RJ assisted in critically revision of the manuscript. All authors read and approved the final manuscript.

## Pre-publication history

The pre-publication history for this paper can be accessed here:

http://www.biomedcentral.com/1471-2458/13/994/prepub
